# Behaviorally relevant frequency selectivity in single- and double-on neurons in the inferior colliculus of the Pratt’s roundleaf bat, *Hipposideros pratti*

**DOI:** 10.1371/journal.pone.0209446

**Published:** 2019-01-02

**Authors:** Ziying Fu, Guimin Zhang, Qing Shi, Dandan Zhou, Jia Tang, Long Liu, Qicai Chen

**Affiliations:** 1 School of Life Sciences and Hubei Key Lab of Genetic Regulation & Integrative Biology, Central China Normal University, Wuhan, China; 2 College of science, National University of Defense Technology, Changsha, China; Universidad de Salamanca, SPAIN

## Abstract

Frequency analysis is a fundamental function of the auditory system, and it is essential to study the auditory response properties using behavior-related sounds. Our previous study has shown that the inferior collicular (IC) neurons of CF-FM (constant frequency-frequency modulation) bats could be classified into single-on (SO) and double-on (DO) neurons under CF-FM stimulation. Here, we employed Pratt's roundleaf bats, *Hipposideros pratti*, to investigate the frequency selectivity of SO and DO neurons in response to CF and behavior-related CF-FM sounds using *in vivo* extracellular recordings. The results demonstrated that the bandwidths (BWs) of iso-frequency tuning curves had no significant differences between the SO and the DO neurons when stimulated by CF sounds. However, the SO neurons had significant narrower BWs than DO neurons when stimulated with CF-FM sounds. *In vivo* intracellular recordings showed that both SO and DO neurons had significantly shorter post-spike hyperpolarization latency and excitatory duration in response to CF-FM in comparison to CF stimuli, suggesting that the FM component had an inhibitory effect on the responses to the CF component. These results suggested that SO neurons had higher frequency selectivity than DO neurons under behavior-related CF-FM stimulation, making them suitable for detecting frequency changes during echolocation.

## Introduction

FM (frequency modulation) sounds are mainly used by bats preying in open space environments, while CF-FM (constant frequency-FM) sounds are employed by bats (called CF-FM bats) as echolocation signals for hunting in relatively complex environments, such as forests and brushwoods [1]. The auditory scene of CF-FM bats consists of echoes from targets and surrounding objects, such as trees, grasses, and land features, as well as calls from conspecifics and other animals [2]. Furthermore, the call frequency of bats could be changed during flying, which is known as Doppler shift [3, 4]. Therefore, these bats have to deal with complex sound signals in heavy background noise conditions. In order to adapt to these complicated conditions, CF-FM bats have evolved some auditory system mechanisms, including an auditory fovea in the cochlea and Doppler shift compensation area in the auditory cortex, which are responsible for fine frequency analysis [5–7].

In addition to the functional adaptations of the auditory systems, the echolocation sound itself is also considered to be essential for sound processing. CF-FM bats produce echolocation sounds that consist of a rather long CF component (5–80 ms, depending upon the species) invariably terminated by a brief FM component (~1–5 ms). Behavioral studies have demonstrated that the CF component is mainly involved in velocity information processing [8–11], while the FM component is responsible for detecting distance information [12–13]. A recent study on FM bats (Mexican free-tailed bats, *Tadarida brasiliensis mexicana*) found that the bats emitted more quasi-CF-FM sounds in the presence of broadband or band-limited noise environments, whereas they utilize a similar number of FM and CF-FM sounds for echolocation in areas with no background noise [14], suggesting that CF-FM sounds are more suitable for acquiring information under noisy conditions.

Physiological studies using CF-FM sounds as sound stimulation have revealed that cochlear microphonic (CM) and summated neural responses (N1, NLL) evoked by the offset of CF sounds (e.g., CM-aft, N1-off, NLL-off), are suppressed by the FM sound components [15–16]. In addition, according to *in vivo* single cell extracellular recording studies, the addition of an FM component to the CF sounds enhances the duration and intensity sensitivity of the inferior collicular (IC) neurons [17–18]. Notably, the IC neurons are discharged as single-on (SO) or double-on (DO) responders when stimulated with CF-FM sounds. The SO neurons only discharge in response to the onset of the CF component, while DO neurons discharge in response to the onset of both CF and FM components [19]. Further studies have shown that DO neurons have relatively shorter response latencies and recovery cycles under CF-FM stimulation conditions [20–21]. Because the bats will increase the emitted signals repetition rate as they approach the target, which makes their auditory system have to deal with fast sound signals. Therefore, these results suggested that these neurons may be suitable for target range and character determination during the terminal phase of bat’s hunting because they have remarkable response to the FM component and short recovery cycle. Further studies on the delay tuning of these neurons are needed to confirm this hypothesis. *In vivo* intracellular recordings suggested that post-spike hyperpolarization (PSH) is a potential mechanism for the formation of SO and DO neurons [22]. Besides the sound properties, the sound sequences are also important for signal processing in the superior colliculus [23], the auditory cortex [24–25], and the IC [26]. In Seba's short-tailed bat, *Carollia perspicillata*, cortical analysis revealed sharper temporal and object tuning when studied with natural echolocation sequences [24–25], while collicular results demonstrated that natural echolocation sequences can enhance the signal-to-noise ratio of the spiking activity in IC neurons [26]. Therefore, in order to better predict the response behavior of these neurons in a natural environment, the study of the auditory response properties of CF-FM bats using CF-FM sounds is necessary. Hence, the CF-FM sounds are called behavior-related sounds hereafter.

**Fig 1:**
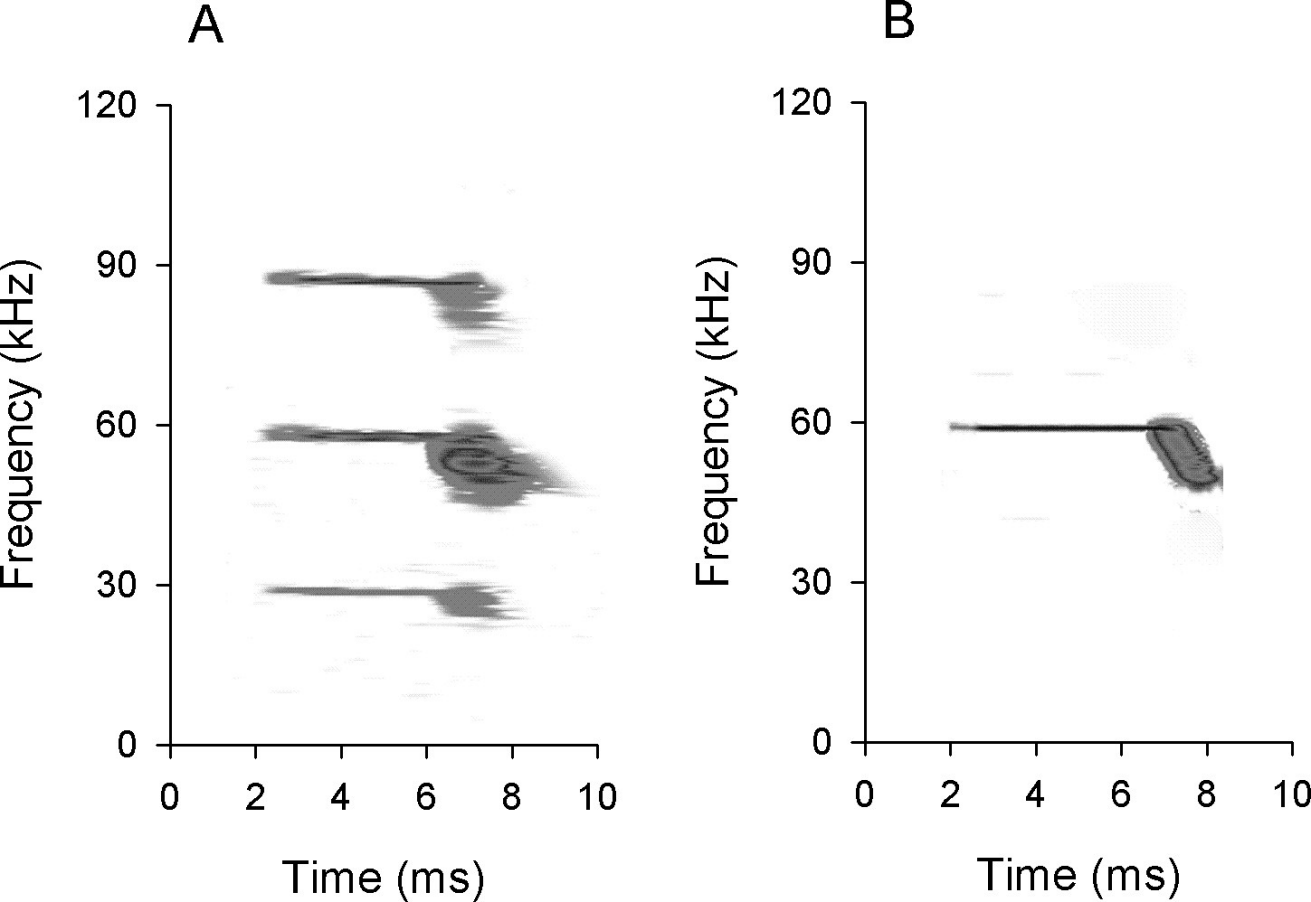
The spectrogram of representative echolocation call of *Hipposideros pratti* (A) and CF-FM stimulus used in our present study (B). The echolocation call included 3 harmonics (H1-H3), and the CF component of these harmonics were 28.9 kHz (CF1), 58.9 kHz (CF2) and 88.5 kHz (CF3).

Frequency analysis is the most fundamental problem in the hearing process [27]. The central auditory system of CF-FM bats contains an overrepresentation of neurons tuned in the foveal frequency range, which is the domain of expanded frequency representation on the basilar membrane. Furthermore, these neurons are sharper tuned relative to the neurons in the non-foveal frequency ranges [11]. Our previous study revealed that preceding behavior-related CF–FM sounds could increase the accuracy of frequency analysis in IC neurons [28]. However, the natural behavior-related sounds effect on frequency selectivity of IC neurons, and the difference of frequency selectivity between SO and DO neurons, remain unclear. Therefore, we addressed these questions using the CF-FM bat, *Hipposideros pratti*. During hanging, the echolocation signals of the bats included a CF and followed by a FM component, and usually had three harmonics (Fig 1A). The CF component of first, second and third harmonics, CF1-CF3, was 31.2±1.6 (26.3–38.2), 60.3±0.7 (59.3–60.9) and 89.2±1.3 (84.9–91.0), respectively. The terminal frequency of the FM component (tFM) of the first, second, and third harmonics, tFM1-tFM3, was 22.7±2.2 (18.8–27.8), 45.4±1.4 (42.7–48.8) and 82.6±3.3 (74.1–86.5), respectively. The FM component dropped about 20% in frequency from the CF component (see S1 Table for detail). We report here that the frequency selectivity could be modulated by the FM component, and that SO neurons have sharper frequency selectivity than DO neurons when stimulated with behavior-related CF-FM sounds. The possible neural mechanism underlying this frequency selectivity sharpening was also studied using *in vivo* intracellular recordings.

## Material and methods

### Ethics statement

All experiments were conducted with the approval of the Institutional Animal Care and Use Committee of Central China Normal University, Wuhan, Hubei, PRC (Permit Number: ccnu2017640-0066). All The surgeries were performed under sodium pentobarbital anesthesia, and all efforts were made to minimize suffering.

### Animals and experimental set up

Fifteen Pratt's roundleaf bats, *Hipposideros pratti* (8 males and 7 females; body weight range: 40.5–56.0 g), caught in a cave (N: 29°26'0.32''; E: 114°01'20.49'') near Xianning City in Hubei province of China, were used in this study. The bats were captured using a ground-level net. A mist net (2.0 m×3.0 m) was opened for 6 h from dusk until midnight (mist net was inspected every 10 min). All bats were housed socially in an animal room (dimensions: 3.0 m×3.0 m×3.0 m) and were exposed to local photoperiod and constant temperature (28–30°C) and humidity (>60% rel. humidity). The bats constantly had free access to water and food (mealworms). The bats were examined daily for any sign of weakness, such as empty stomach or slow response to hand-holding. Bats in poor physiological condition were excluded. The Forestry Department of Hubei Province provided permission to conduct the study on this site. No specific permissions were required for our research because *H*.*pratti* is not considered an endangered or protected species. The surgical procedures applied were the same as described in our previous studies [19, 22]. Briefly, the flat head of a 1.8-cm nail was glued onto the exposed skull of each Nembutal-anesthetized bat (45–50 mg/kg b.w.) with acrylic glue and dental cement, one or two days before the recording session. Exposed tissues were treated with an antibiotic (Neosporin) to prevent infection. The bat was administered with the neuroleptanalgesic, Innovar-Vet (Fentanyl: 0.04 mg/kg b.w.; Droperidol: 2 mg/kg b.w.) and placed inside a bat holder that was suspended by an elastic sling inside a custom-made double-wall sound-proof room (temperature: 28–30°C).

After fixing the bat’s head with a set screw, small holes (200–500 μm) were made in the skull above the IC for orthogonal insertion of 2 M NaCl (electric impedance: 5–10 MΩ) or 1 M tri-potassium citrate (electric impedance: 23–104 MΩ) glass pipette electrodes to record extracellular or intracellular sound-activated responses. The glass pipette electrodes were pulled from a single glass barrel with a thick wall (outer-diameter: 1.5 mm), and a thin filament with a microelectrode puller (Sutter, P-97, USA) in the interior cavity. Additional doses of Innovar-Vet were administered during the later phases of recordings when the bats showed signs of discomfort, as indicated by restless minor body movements. A local anesthetic (Lidocaine) was applied to the open wound area to reduce any possible pain. The recording depth was measured from the scale of a microdrive (model 640, David Kopf Instrument; SM-21, Narishige). A common electrode was placed at the nearby temporal muscles.

### Generation of behavior-related CF-FM sounds

For acoustic stimulation, continuous sine waves from a function generator (GFG-8016G, Good Will Inst Co., Ltd) were converted into pure tone pulses (5 with 0.5 ms rise-decay times, delivered at 2 pulses per second, referred to as CF sound) by a custom-made tone burst generator driven by a stimulator (Model SEN-7203, Nihon Kohden Co; Master-8, A.M.P.I.). The FM sound (2 ms with 0.5 ms rise-decay time) was generated by a function generator (3320A, HP) using linear ramp signals. The tone pulses were then amplified after passing a decade attenuator (LAT-45, Leader) before they were fed into a small loudspeaker (AKG model CK 50, 1.5 cm in diameter, 1.2 g, frequency response 1–100 kHz). The loudspeaker was placed 30 cm away from the bat’s ear and at 30°contralateral to the recording site. Calibration of the loudspeaker was conducted with a 1/4-inch microphone (4939, B&K) placed at the bat’s ear using a measuring amplifier (2610, B&K). The output of the loudspeaker was expressed in decibel sound pressure level (dB SPL) in reference to 20 μPa root mean square. A frequency-response curve of the loudspeaker was plotted to determine the maximal available sound amplitude at each frequency. The maximal stimulus amplitude ranged from 110 to 125 dB SPL between 10 and 80 kHz but dropped off sharply to 80 dB SPL at 100 kHz thereafter.

The CF-FM sound, which mimics the echolocation signal emitted by *H*. *pratti*, is called behavior-related sound signal in the present study, was generated by means of a linear voltage ramp from the function generator driven by the same electronic switch. A CF sound was first generated. Then, the onset of the linear voltage ramp was adjusted and synchronized at the time equal to the CF component of the CF sound such that the amplitude was not changed at the boundary between the CF and FM components, while the frequency of the CF sound (referred to as CF frequency) was reduced 20% within the FM portion (Fig 1B). That is, the end frequency of the 2-ms FM component was 80% of the CF frequency. We swept the FM component for about 20% because our recorded echolocation signals showed the FM component was downward about 20%.

### *In vivo* extracellular and intracellular recordings

**Fig 2:**
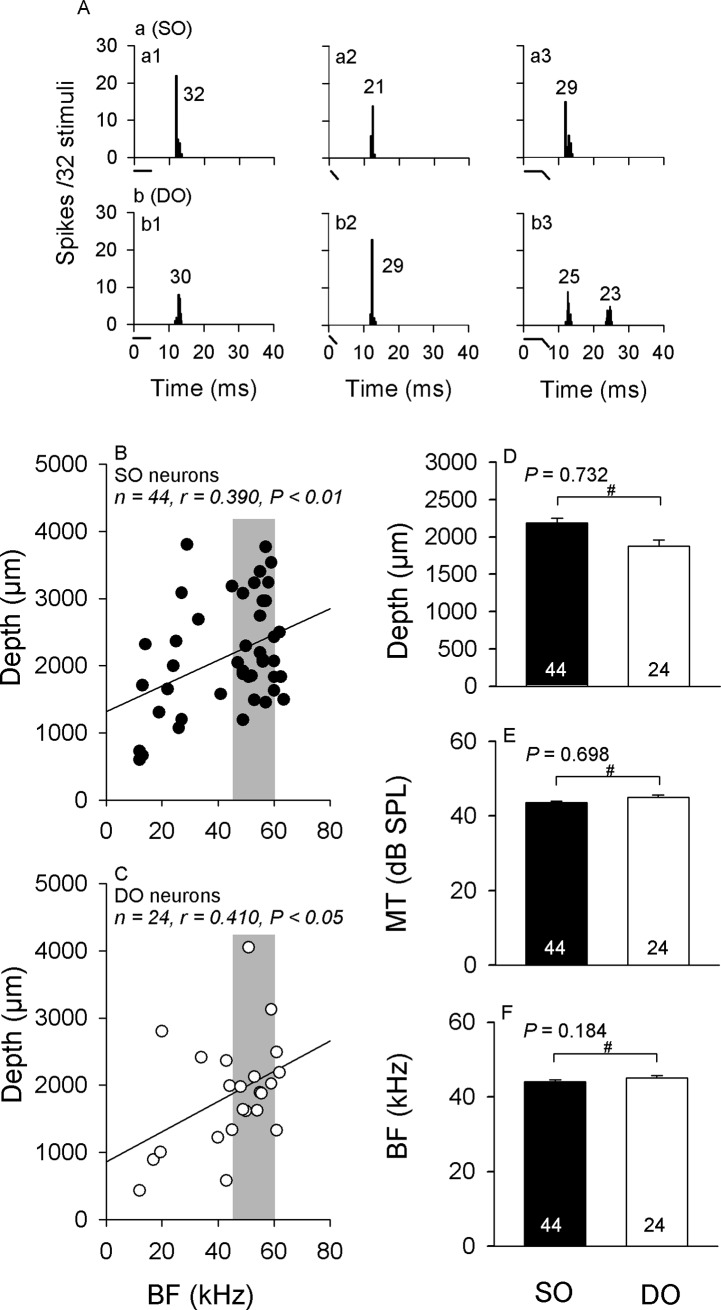
Basic properties of the SO and DO neurons. (A): SO and DO neurons. The PSTHs show the discharge pattern and number of impulses in the SO and DO neurons. Whereas both neurons discharged impulses to CF, FM and CF-FM sounds (Aa), only the DO neuron discharged impulses to the FM component of the CF-FM sound (Ab3). A sketch of each sound stimulus is shown below the abscissae. (B) and (C): Scatter plots of the distribution of BFs in relation to the recording depth of (B) SO and (C) DO neurons. The linear regression line is shown by a solid line. r is the correlation coefficient of the distribution. The grey rectangle indicates the frequency range of the bat’s second harmonic. (D): Average recording depth, (E): MT, and (F): BF of SO (solid bar) and DO (empty bar) neurons. # *P* >0.05.

For *in vivo* extracellular recordings, upon isolation of a neuron with 5-ms CF sound, its sound-activated response was amplified (ISO-DAM, WPI) before being sent to an oscilloscope (TDS210, Tek) and an audio monitor (AM9, Grass). The neuron’s threshold at each responsive frequency was audio-visually determined by changing the sound amplitude, which on average elicited 50% response probability. The sound frequency that elicited the neuron’s response at the lowest amplitude was defined as the best frequency (BF). The threshold at the BF was defined as the minimal threshold (MT). The neuron’s response data was further processed for acquisition of peri-stimulus-time histograms (PSTH) (bin width: 250 μs) to 32 sound presentations (Fig 2A). The number of impulses in each histogram was used to quantify the neuron’s response under each sound condition.

**Fig 3:**
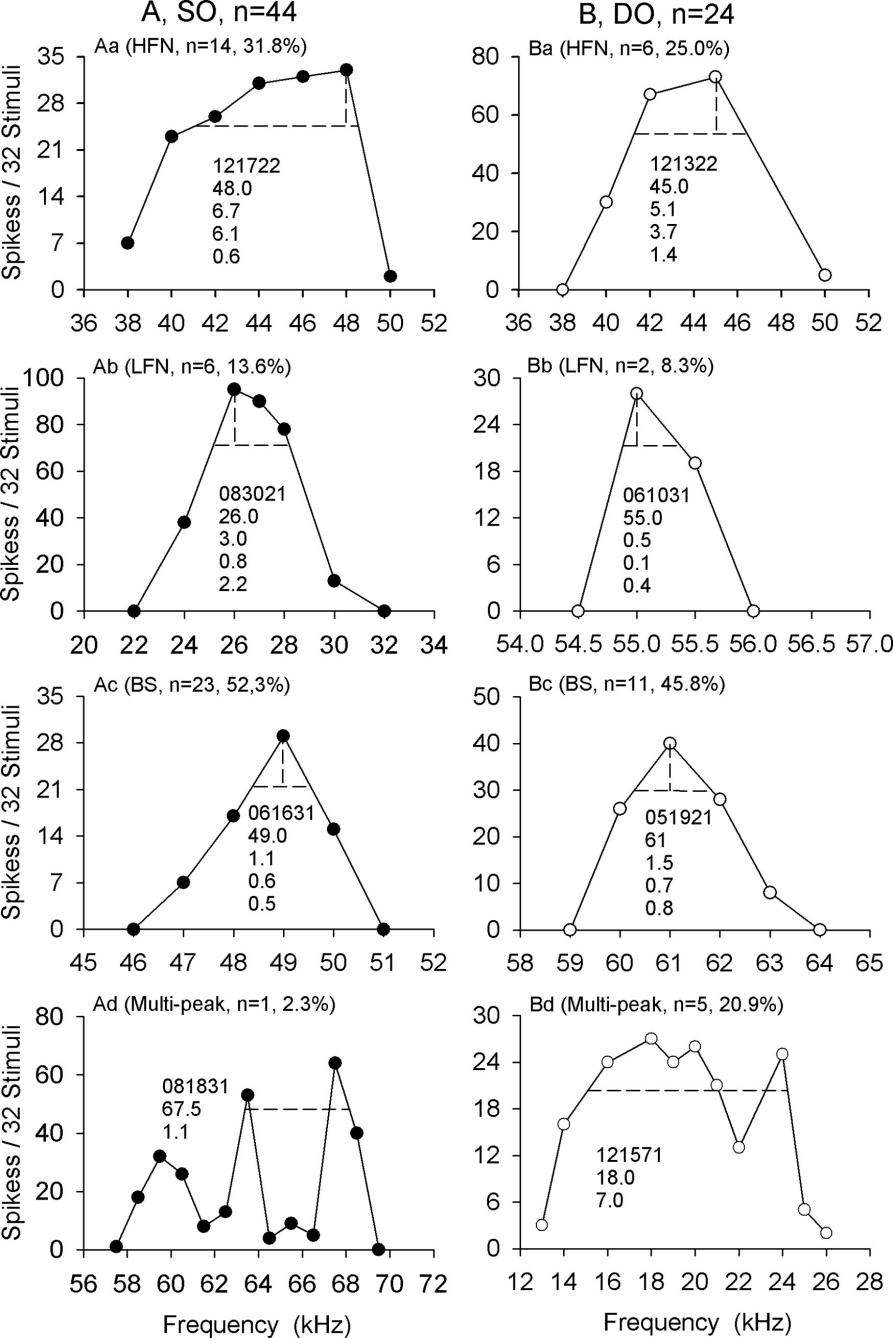
The iso-level frequency tuning curve (FTC) stimulated by CF sounds. Iso-level FTC in (A) DO and (B) SO neurons; (a): representative HFN, (b): LFN, (c): BS, and (d): multi-peak. The number and percentage of each type of iso-level FTC are shown as n and %, respectively. The numbers in each figure represent the data number of each neuron; BF, BW, BWL, and BWH are shown in order from top to bottom.

The frequency selectivity of the IC neurons was studied by measuring the iso-level frequency tuning curve (FTC) with the number of impulses discharged to the stimulation at selected frequencies. Usually, selected frequencies were in 1 kHz step size decreasing or increasing from each neuron’s BF until the neuron had no response. However, if a neuron had very sharp frequency tuning, a 0.5 or 0.1 kHz step size was used. It should be emphasized that for the iso-level FTC of DO neurons stimulated with CF-FM sounds, only the impulses elicited by the CF component were taken into consideration, and the response duration was restricted to 5 ms (CF component duration) starting from the first spike latency (minimum spike latency observed across 32 sound presentations). The iso-level FTCs under CF and CF-FM stimulation were determined randomly. The frequency selectivity was expressed with a band-width (BW) of each iso-level FTC at 75% of maximal number of impulses (Fig 3). An iso-level FTC with a small BW has sharper frequency selectivity than an iso-level FTC with a large BW. The neuron’s BW obtained under different stimulation conditions or between different groups was then quantitatively examined and statistically compared using repeated measures. When the sound stimulation was switched to CF-FM stimuli from CF stimuli, the BWs could be increased (the BW under CF-FM stimulation was more than 20% broader than the BW under CF stimulation), decreased (the BW under CF stimulation was more than 20% broader than the BW under CF-FM stimulation) or unchanged (the difference in the BW under CF and CF-FM stimulation was no more than 20%). The BW change was also quantified and was defined as the BW difference value under CF-FM and CF stimulation condition, and divided by the BW under CF stimulation condition. Therefore, higher change rate represents larger BW change, and a positive change rate indicates that the BW under CF-FM stimulation was higher than the BW under CF stimulation, and vice versa.

For the *in vivo* intracellular recordings, the depth referring to the surface of the IC of each recorded neuron was obtained from the remote controller of a microelectrode drive (SM-21, Narishige). The action potentials (APs) of IC neurons evoked by tone burst were recorded (Axon Axopatch 900A, AXON). The sound frequency that elicited most APs at given amplitude was defined as the BF, and the amplitude elicited 50% response probability was defined as MT. Each isolated IC neuron was stimulated 8 times with CF and behavior-related CF-FM sounds. The post-spike hyperpolarization latency (PSHL) was defined as the first time when the membrane potential reached 10% PSH amplitude relative to the onset of sound stimulation, and the excitatory duration (ED) was defined as the time course during which the membrane potential was higher than the averaged resting potential before the spikes.

### Statistical analysis

All data obtained are presented as mean ± SD, and were processed and plotted using Sigmaplot 10.0. When the F test was not significant and homogeneity of variance was assumed, Student’s t-test was performed to compare results between two experimental groups. When the F test was significant and homogeneity of variance was not assumed, Welch’s t-test was used instead. The paired t-test was used to compare results from two experimental groups of the same cells. In all tests, *P* < 0.05 was considered statistically significant.

## Results

### Basic properties of SO and DO neurons

A total of 125 IC neurons were isolated by extracellular recordings. Because of restrictions of recording conditions and time, the frequency selectivity tested completely with both CF and CF-FM sounds was assessed only in 68 of them. These neurons discharged as onset responders when stimulated with CF or FM sounds (Fig 2Aa1, a2, b1, b2). Different form previous studies on CF-FM bats [29], we did not find any on-off responders under our previous sound stimulation condition. The most possible reason for the lack of on-off responders might be the sound frequency, since a recent study showed that the IC neurons responded with on-off pattern at the resting frequency, whereas at the resonance frequency these neurons responded with a phasic-like pattern [30]. In accordance with our previous studies [19, 22], the IC neurons discharged as SO and DO responders when they were stimulated with behavior-related CF-FM sounds. In addition, SO neurons only discharged impulses to the onset of CF, FM and CF-FM stimuli (Fig 2Aa, a1-a3), while DO neurons also only discharged impulses to the onset of CF and FM sounds (Fig 2Ab, b1-b2), but also discharged to the onset of the CF and FM components of the CF-FM stimuli (Fig 2Ab, b3). Please note that the response latency of the DO neurons to the FM component was longer than to the CF component for the responses to CF-FM stimuli (Fig 2Ab, b3), possibly because of the inhibition caused by the CF component. Linear regression analysis showed that the BF of both the SO (n = 44, r = 0.390, *P* <0.01; Fig 2B) and the DO (n = 24, r = 0.410, *P* <0.05; Fig 2C) neurons increased with the recording depth. Statistical results revealed that there were no significant differences in the recording depth [603–4380 (2190.5 ± 57.8) vs. 428–4050 (1879.3 ± 78.3) μm, *P* = 0.732; Fig 2D], MT [32–101 (43.5 ± 0.4) vs. 34–81 (45.0 ± 0.6) dB SPL, *P* = 0.698; Fig 2E] and BF [12.0–63.5 (44.1 ± 0.5) vs. 12.0–62.0 (45.0 ± 0.6) kHz, *P* = 0.184; Fig 2F] between SO and DO neurons, respectively. These results indicate that SO and DO neurons are topologically organized and discretely distributed across the dorsal-ventral axis of the IC neurons, which is similar to our previous findings in the same species [22] and in other CF-FM bat species [19].

### Iso-level FTC of SO and DO neurons

The iso-level FTC of the IC neurons was first studied under 5- ms CF sound stimulation. The number of impulses were constantly the largest when stimulated with BF sounds, and the responses of most neurons to alterations in the sound frequency from the BF (F) decreased monotonically, forming an “inverted V” shape FTC for both SO and DO neurons (Fig 3Aa-c and 3Ba-c). Meanwhile, a small number of neurons also displayed peak responses to other frequencies beside the BF, forming a multi-peak FTC (Fig 3Ad and 3Bd). For the “inverted V” shape FTCs, the BW was calculated as the crossings in the TC at 75% of the maximum spiking observed at the BF. We calculated the half the BW from the BF at both the low frequency side (BWL) and the high frequency side (BWH). These neurons were classified into three types based on the relative values obtained for BWL and BWH, i.e. the high frequency side narrower (HFN), the low frequency side narrower (LFN), and the both side symmetry (BS) neurons. The neurons were defined as HFN (Fig 3Aa and 3Ba) when the BWL was more than 20% higher than the BHL, LFN neurons (Fig 3Ab and 3Bb) when the BWH was more than 20% higher than the BWL, and BS (Fig 3Ac and 3Bc) neurons when the difference between BWH and BWL was no more than 20%. Notably, the BS neurons bear the largest percentage of both SO (52.3%) and DO (45.8%) neurons, followed by the HFN neurons (31.8% SO neurons and 25.0% DO neurons). It is worth mentioning that DO neurons (20.9%) displayed relatively higher percentage of multi-peak FTC relative to the SO neurons (2.3%).

When stimulated with behavior-related CF-FM sounds, which was generated by means of a linear voltage ramp from the function generator driven by the same electronic switch (please see Material and methods for detail), half of the HFN (7/14) and LFN (3/6) in SO neurons changed into BS FTCs, while most of the BS in SO neurons remained unchanged (16/23) and other neurons were altered into LFN (4/23) or multi-peak (3/23) FTC neurons. Therefore, the percentage of BS (from 52.3% to 61.4%), LFN (from 13.6% to 20.4%), and multi-peak (from 2.3% to 9.1%) FTC neurons increased, at the cost of a great reduction in the percentage of HFN FTC neurons (from 31.8% to 9.1%), making the HFN FTC neurons more predominant under CF-FM sound stimulation conditions (Table 1). However, the percentage of DO neurons’ iso-level FTC was similar between the CF and the CF-FM stimulation conditions; even though it was slightly altered due to changes in the stimulation conditions (Table 2).

**Table 1:** The iso-level frequency tuning curve (FTC) types under CF and CF-FM stimulation of SO neurons.

	CF	CF-FM				
		HFN	LFN	BS	MP	
HFN	14(31.8)	3	3	7	1	
LFN	6(13.6)	1	2	3	0	
BS	23(52.3)	0	4	16	3	
MP	1(2.3)	0	0	1	0	
Total	44	4(9.1)	9(20.4)	27(61.4)	4 (9.1)	

SO: single-on; CF: constant frequency; FM: frequency modulation; HFN: high frequency side narrower; LFN: lower frequency side narrower; BS: both side symmetry. The values represent the number of neurons and the numbers in parenthesis indicate percentage.

**Table 2:** The iso-level frequency tuning curve (FTC) types under CF and CF-FM stimulation of DO neurons.

	CF	CF-FM			
		HFN	LFN	BS	MP
HFN	6(25)	2	0	3	1
LFN	2(8.3)	0	1	1	0
BS	11(45.8)	4	0	5	2
MP	5(20.9)	1	1	2	1
Total	24	7(29.2)	2(8.3)	11(45.8)	4(16.7)

DO: double-on; CF: constant frequency; FM: frequency modulation; HFN: high frequency side narrower; LFN: lower frequency side narrower; BS: both side symmetry. The values represent the number of neurons and the numbers in parenthesis indicate percentage.

### BWs of SO and DO neurons

**Fig 4:**
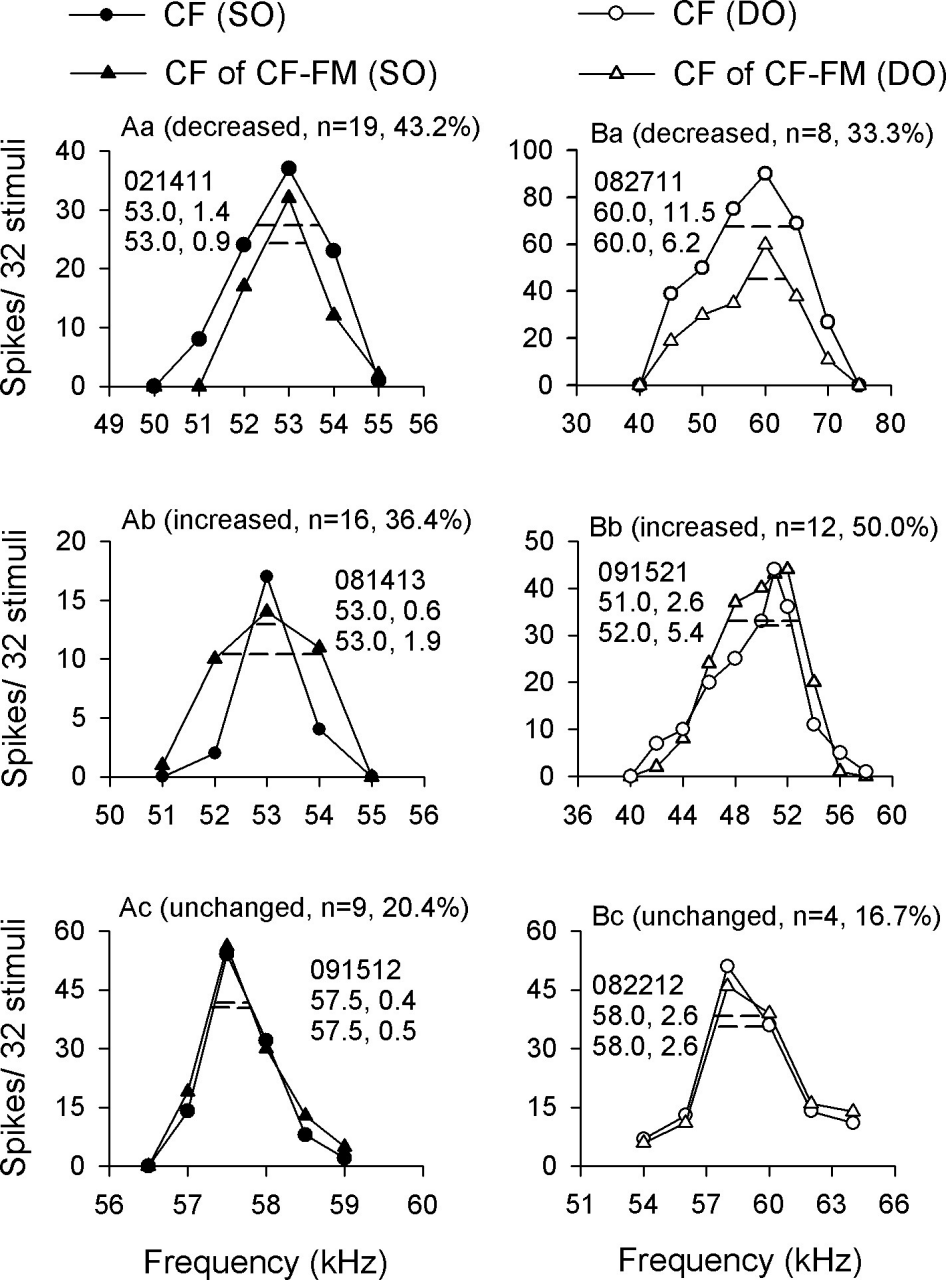
The BWs changes after switching the CF to CF-FM sounds. (A): SO and (B) DO neurons. The BWs were (a) decreased, (b) increased, and (c) did not change, after switching the CF to CF-FM sounds. The number and percentage of each type of change are shown as n and %, respectively. The numbers in each figure represent the data number of each neuron; BF and BW under CF stimulation, BF and BW under CF-FM stimulation.

In order to quantitatively analyze the effect of the FM component on the frequency selectivity of SO and DO neurons, we calculated the BWs in these neurons. Compared to the BWs under CF stimulation, the BWs under CF-FM stimulation were decreased, increased or unchanged for both SO (Fig 4Aa-c) and DO (Fig 4Ba-c) neurons (see methods for detail). In detail, the percentage of decreased, increased and unchanged BWs in SO neurons were 43.2% (Fig 4Aa), 36.4% (Fig 4Ab), and 20.4% (Fig 4Ac), respectively; and the percentage of decreased, increased and unchanged BWs in DO neurons were 33.3% (Fig 4Ba), 50.0% (Fig 4Bb), and 16.7% (Fig 4Bc), respectively. Please note that the BW change occurred conjointly with a change in the number of spikes. The FM components could decrease the number of spikes elicited by CF components when its frequency was equal to BF for both BW decreased and increased neurons (Fig 4Aa-b and 4Ba-b). However, when the frequencies of CF components were beside the BF, the BW decreased neurons also had decreased number of spikes to CF components (Fig 4Aa and 4Ba) while the BW increased neurons could have slightly decreased or even increased number of spikes to CF components (Fig 4Ab and 4Bb). The BW unchanged neurons had similar number of spikes to CF components of CF-FM sounds and CF sounds (Fig 4Ac and 4Bc).

**Fig 5:**
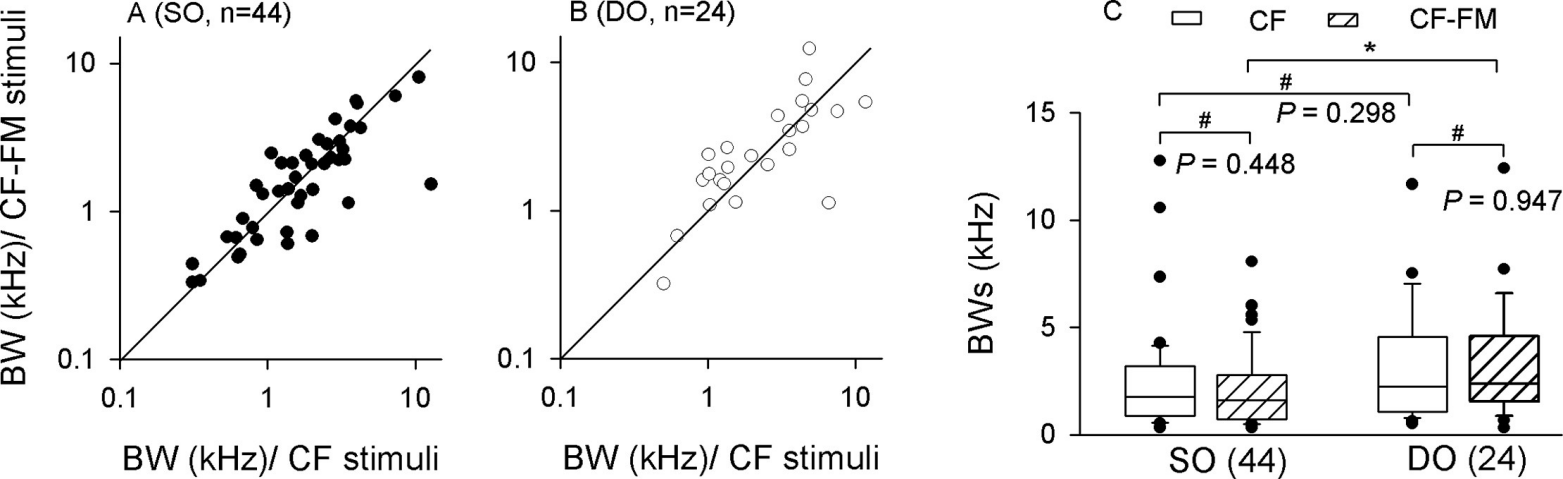
BW distribution of SO and DO neurons. Scatter plots showing the distribution of BWs under CF and CF-FM stimulation for (A) SO and (B) DO neurons. The solid line represents the equal-value diagonal line and the dashed line is the 0.5-kHz difference line. (C): Box plots show BWs under CF and CF-FM stimulation for SO and DO neurons. # *P* >0.05; * *P* <0.05.

Scatter plot analysis was performed to illustrate the distribution of BWs under CF and CF-FM stimulation (Fig 5A and 5B). Most of the SO neurons had BWs below 10 kHz under CF and CF-FM stimulation (Fig 5A). Although most SO neurons had similar BWs under CF and CF-FM stimulation (Fig 5A; filled circles around the equal line), there were still some neurons that had relative narrower BWs under CF-FM stimulation (Fig 5A; filled circles far below the equal line). The average BWs of SO neurons had no significant difference between CF and CF-FM stimuli (2.46 ± 0.24 vs 2.12 ± 0.18 kHz, respectively; *P* = 0.448; Fig 5C). Similar to SO neurons, DO neurons also had the majority of BWs below 10 kHz under CF and CF-FM stimulation (Fig 5B). However, there were few neurons which displayed higher BW in response to CF-FM stimuli (Fig 5B; open circles far above the equal line). The average BWs of DO neurons were almost the same under both CF and CF-FM stimulation (3.14 ± 0.42 vs. 3.19 ± 0.39 kHz, respectively; *P* = 0.947; Fig 5C). Statistical analysis showed that the BWs of SO and DO neurons had no significant difference between them under CF stimulation (2.46 ± 0.24 vs. 3.14 ± 0.42 kHz, respectively; *P* = 0.298; Fig 5C), but the BWs of DO neurons were significantly higher than in SO neurons under behavior-related CF-FM stimulation (2.12 ± 0.18 vs. 3.19 ± 0.39 kHz, respectively; *P* < 0.05; Fig 5C).

**Fig 6:**
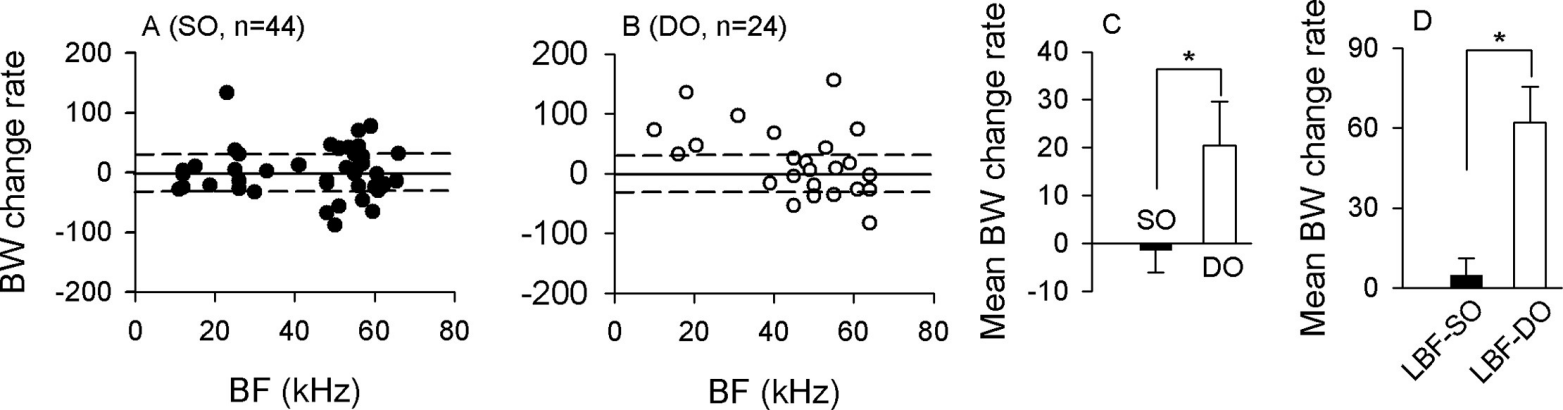
BW Change rate of SO and DO neurons. The distribution of the BWs change rate against BF for (A) SO and (B) DO neurons. The solid line is the “zero line”, indicating that no change was observed in the BWs under CF and CF-FM stimulation; and the dashed line represents the 30% change rate line. (C): Mean BWs change rate of SO and DO neurons. (D): Mean BWs change rate of low BF SO and low BF DO neurons. LBF SO, low BF SO neurons; LBF DO, low BF DO neurons. * *P* <0.05.

We further studied the possible relation between BWs changes and BFs in SO and DO neurons. The BW change was quantified by the change rate (see the methods for detail). Fig 6A shows the distribution of BW change rate in relation to the BF of SO neurons. Most data points with BF from 10 to 70 kHz were distributed around the “zero line”, and some neurons with BF between 45 and 60 kHz, which were tuned at the second harmonic of the echolocation signal of this bats, had higher BWs change rates and displayed a BWs change rate higher or lower than the 30% change rate line (Fig 6A). In contrast, most of the DO neurons had BWs around the “zero line”, with some neurons having relatively higher change rate as indicated by the data points above the 30% change rate line (Fig 6B). Statistical analysis showed that the change rates were significantly higher in DO neurons than in SO neurons (Fig 6C). It is interesting to notice that the DO neurons with BF lower than 45 kHz (low BF DO neurons) had relatively higher change rate the SO neurons (low BF SO neurons) (Fig 6A, 6B and 6D), which probably indicate that these low BF DO neurons might suitable for processing of communication calls.

### BF change of SO and DO neurons under CF and CF-FM stimulation

**Fig 7:**
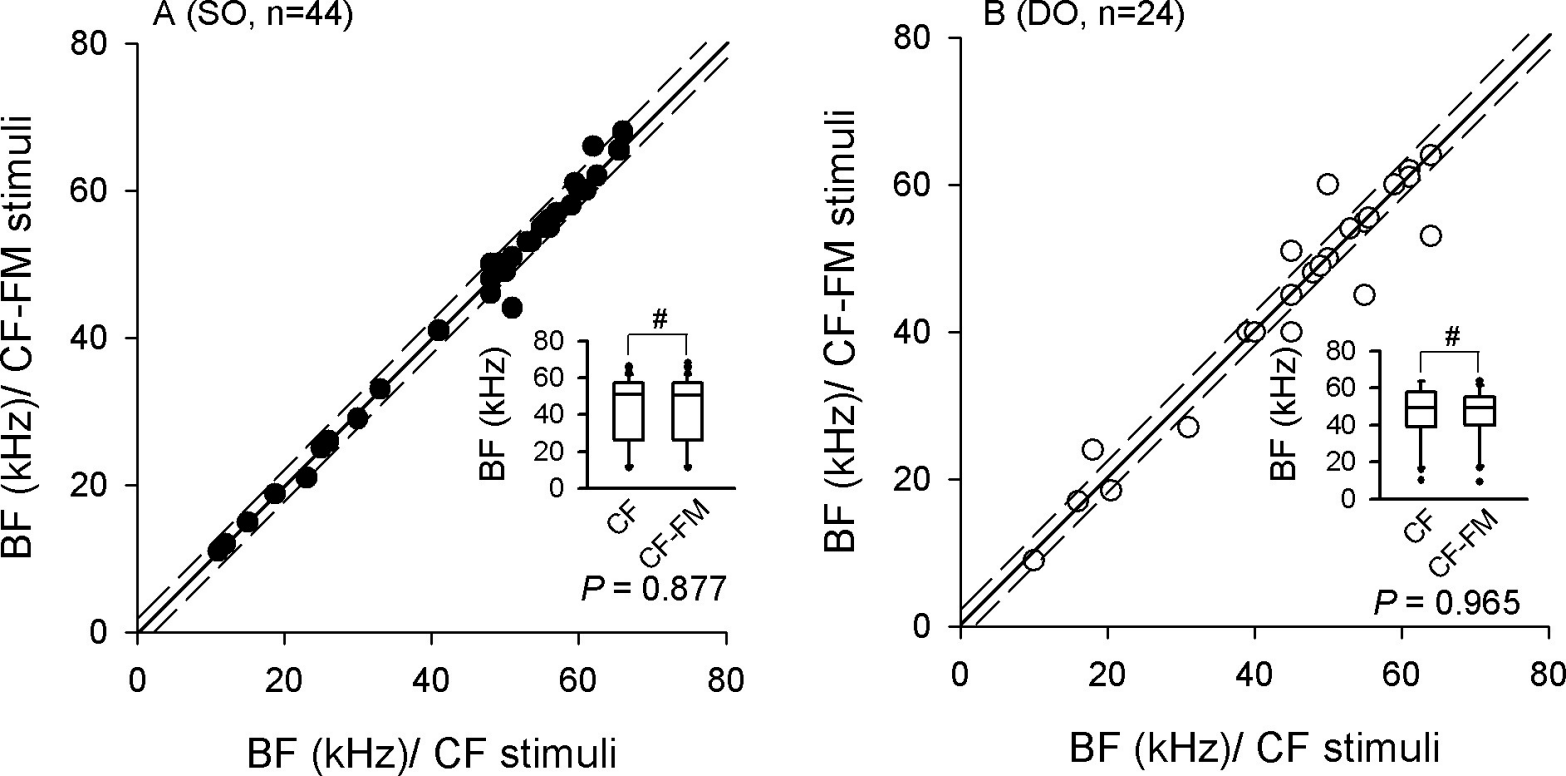
Scatter plots of BFs under CF and CF-FM stimulation for (A) SO and (B) DO neurons. The solid line represents the equal-value diagonal line and the dashed line is the 2-kHz difference line. The inset box plots show that the BFs under CF and CF-FM stimulation had no significant different between the SO (A inset) and the DO (B inset) neurons.

Besides the BWs change of SO and DO neurons, we also noticed that, while most of the SO and DO neurons had the same BF under CF and CF-FM stimuli (Figs 4Aa-c, 3Ba and 3Bc), there were few neurons that responded with changes in their BF when stimulation was switched from CF to CF-FM sounds (Fig 4Bb). Scatter plots showed that the BFs of most of the SO neurons were similarly distributed under CF and CF-FM stimulation (Fig 7A; elucidated by the filled circles evenly distributed around the equal line and between the 1 kHz difference line), with the only exception of one neuron (Fig 7A). Most of DO neurons also had similar BFs as the SO neurons under CF and CF-FM stimuli, but few neurons had higher BFs under CF (Fig 7B: open circles below the low 1 kHz difference line) or under CF-FM (Fig 7B; open circles above the upper 1 kHz difference line) stimulation (Fig 7B). Statistical analysis showed that there was no significant difference in the BFs between CF and CF-FM stimuli in SO (45.8 ± 2.5 vs. 45.0 ± 2.5 kHz, respectively; *P* = 0.877; Fig 7A, inset) and DO (43.4 ± 3.2 vs. 43.3 ± 3.2 kHz, respectively; *P* = 0.965; Fig 7B, inset) neurons.

### Post-spike hyperpolarization latency and excitatory duration of SO and DO neurons

Our *in vivo* extracellular recording results showed that the FM component could increase the frequency selectivity of some SO neurons, and the responses to CF components were decreased compared to the CF sounds, indicating an inhibitory effect of the FM component on the CF component during CF-FM stimulation. To have an insight on the possible mechanism for this inhibition, we performed *in vivo* intracellular recording in response to CF and CF-FM sounds. A total of 37 IC neurons were isolated, and the BF, MT, and recording depth measurements for these neurons were 27–66 (51.4 ± 5.7) kHz, 44–94 (68.8 ± 12.7) dB SPL, and 1159–4217 (2820.7 ± 497.0) μm, respectively. Statistical analysis demonstrated that these neurons had no significant differences in the BF (51.4 ± 5.7 vs. 44.4 ± 2.8 kHz, respectively; *P* = 0.144) and in the recording depth (2820.7 ± 497.0 vs. 2053.7 ± 363.3 μm, respectively; *P* = 0.103) when compared to neurons recorded extracellularly, suggesting that the neurons isolated extracellularly and intracellularly were located in a similar region.

**Fig 8:**
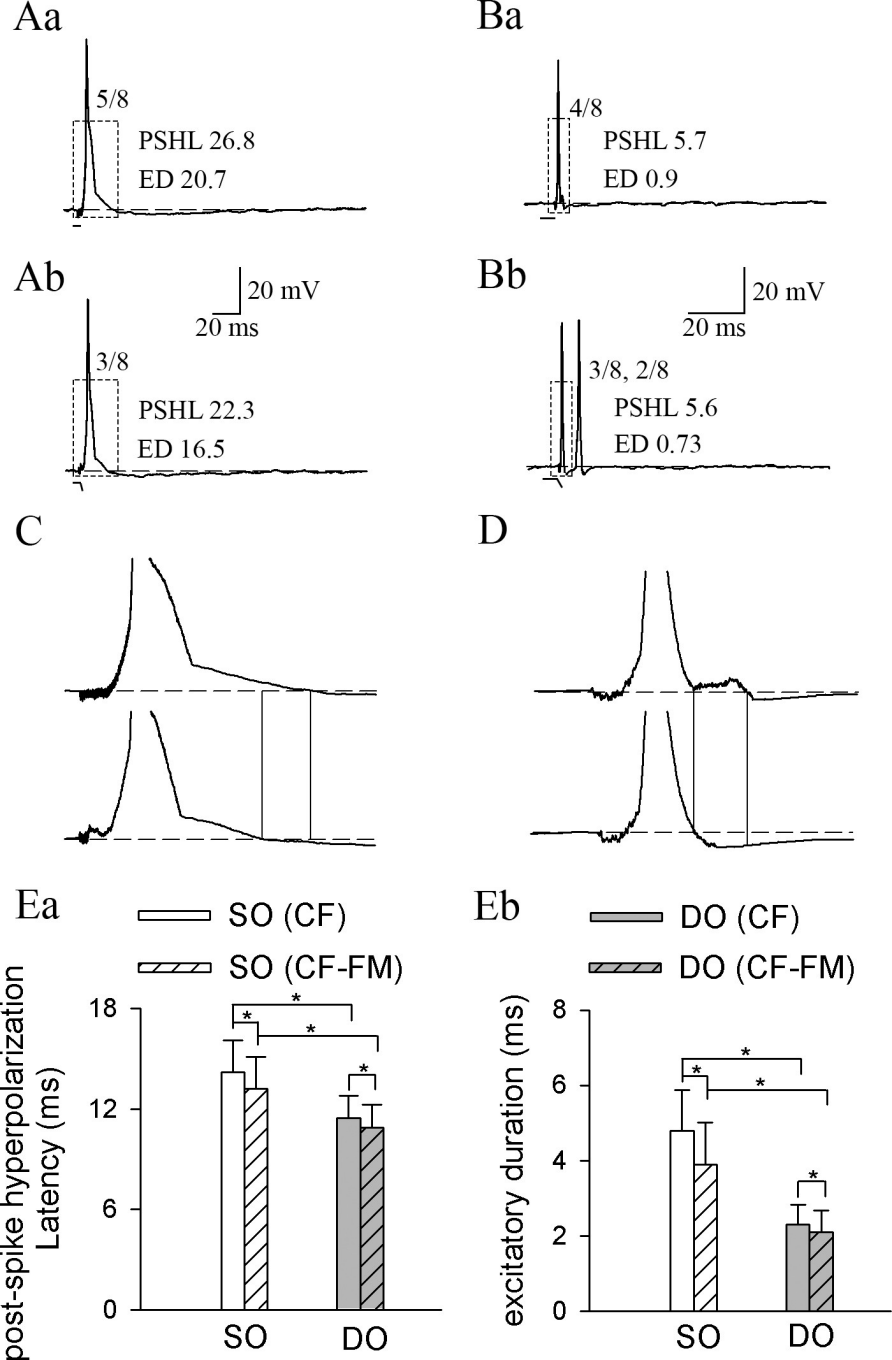
Post-spike hyperpolarization latency (PSHL) and excitatory duration (ED) of SO and DO neurons. (A) and (B): representative responses of (A) SO and (B) DO neurons to (a) CF and (b) CF-FM sounds. The number besides the action potential (AP) firing in each panel represents the total number of APs elicited by various stimuli per eight trials. The number after PSHL and ED in each panel is the average PSHL and ED value of each representative response. A sketch of each sound stimulus is shown below the neuron’s response. (C) and (D): Magnified view of the area highlighted with a dashed line rectangle in the corresponding response traces of (C) SO and (D) DO neurons. (E): comparisons of (a) PSHL and (b) ED under CF and CF-FM stimulation for SO and DO neurons. The PSHL under CF stimulation for SO and DO neurons were 14.2 ± 1.9 and 11.4 ± 1.3 ms; The PSHL under CF-FM stimulation for SO and DO neurons were 13.2 ± 1.8 and 10.9 ± 1.2 ms (Ea). The ED under CF stimulation for SO and DO neurons were 4.8 ± 1.1 and 2.3 ± 0.5 ms; The ED under CF-FM stimulation for SO and DO neurons were 3.9 ± 1.1 and 2.1 ± 0.6 ms (Eb). * *P* <0.05.

The responses to CF and CF-FM sounds were recorded in 19 out of the isolated 37 neurons; 13 were SO neurons (Fig 8A) and 6 were DO neurons (Fig 8B). When stimulated with a sound above the MT, an initial spike or spikes appeared, indicating membrane potential depolarization, followed by a termination signal with PSH (Fig 8A and 8B). Compared to CF stimuli, the CF component of CF-FM stimuli elicited less spikes and a shorter PSHL and ED, for both SO (Fig 8Aa-b and 8C) and DO (Fig 8Ba-b and 8D) neurons. Statistical analysis demonstrated that both SO and DO neurons had significantly shorter PSHL and ED under CF-FM stimulation (Fig 8Ea), and the SO neurons had longer PSHL and ED than DO neurons (Fig 8Eb). These finding indicate that the SO neurons had stronger excitatory input than DO neurons, being consistent with the results of our previous study, which showed that SO neurons had higher firing rates [21]. The different response properties between SO and DO neurons also suggested that they have different morphology, which need future studies to reveal it.

## Discussion

In the present study, we first compared the frequency selectivity of SO and DO neurons under CF and CF-FM stimulation, aiming to reveal the possible modulation mechanism of the FM component on frequency analysis in IC neurons. The results showed that the SO neurons had sharper frequency selectivity than the DO neurons under behavior-related CF-FM stimulation, while both SO and DO neurons had similar frequency selectivity under CF stimulation. *In vivo* intracellular recordings showed that both SO and DO neurons had shorter inhibitory input latency and excitatory input duration in response to CF-FM than CF stimuli, and SO neurons had significantly longer inhibitory input latency and excitatory input duration than DO neurons under both CF and CF-FM stimulation.

### Responses to behavior-related CF-FM sounds

Ethologically relevant sounds are usually complex, and understanding how the brain processes sounds related to the life experiences of an animal is important. The CF-FM bats emit CF-FM sounds during hunting and communicating [31, 32]. Therefore, it is essential to know how the auditory system responds to these behavior-related sounds for investigating the neurophysiology of echolocation and also for the understanding of the processing of complex sounds. An earlier study on collicular evoked responses showed typical response to both the CF and the FM components of simulated behavior-related sounds [33], but the authors did not assess the changes in the duration of the CF or FM components to verify whether the response to FM component was an on- or off-response. Suga and his colleagues observed that the cochlear microphonic (CM) and summated neural responses (N1, NLL), evoked by the offset of the CF sound (e.g., CM-aft, N1-off, NLL-off), were suppressed by the FM component [15–16]. *In vivo* single cell recording in the IC neurons of *Rhinolophus ferrumequinum* demonstrated that some neurons had two separate cluster responses, but they could not clarify whether the later response was attributed to the FM component or to the off-response to the CF component [34]. Our previous work on *Hipposideros armiger* clearly confirmed that the later cluster of responses were triggered by the onset of the FM component, as their response latency increased in parallel with the increasing CF component, but remained nearly constant when the FM component was increased. These neurons were called DO responders, and the neurons that only responded to the onset of the CF component were called SO responders [19]. Further *in vivo* intracellular recording revealed that PSH was the possible mechanism of shaping the response patterns of SO and DO neurons [22]. We also showed that the IC neurons had less responses, higher duration and amplitude selectivity to the CF component, demonstrating that the FM component could suppress the responses to the CF component [17, 18, 21]. Moreover, the present study showed that IC neurons had higher frequency selectivity under behavior-related sounds for SO neurons, which further indicates the inhibitory effect of the FM component. *In vivo* intracellular recordings illustrated that the PSHL and ED were shorter, further supporting that the suppression may be attributed to the inhibitory effect of the FM component (Fig 8).

### Frequency selectivity under behavior-related sounds

The *in vivo* intracellular results showed that the FM component could inhibit the CF responses of both SO and DO neurons, and this inhibition was thought to be the mechanism of sharpening the frequency selectivity. However, the reason why the BWs of some neurons had no change or even increased still remained unclear. In our present study, we used iso-level FTCs and BWs to describe the frequency selectivity. According to our analysis, when a neuron had strong response to its BF and less responses to frequencies beside its BF, the neuron exhibited a narrow FTC and a small BW, indicating high frequency selectivity. For neurons with higher frequency selectivity under CF-FM stimulation, the inhibitory effect of the FM component was more predominant on the frequencies besides their BFs, and vice versa. For neurons with no difference in frequency selectivity under CF or CF-FM stimulation, the inhibitory effect remained similar across the frequencies tested. Our results showed the SO neurons had significant higher frequency selectivity than DO neurons under CF-FM stimulation. Therefore, we speculate that the SO neurons receive more inhibitory input when stimulated with frequencies beside their BFs.

Inhibition is considered to play a crucial role in the regulation of many selectivity properties including frequency selectivity [27, 35]. *In vivo* whole-cell recordings have demonstrated that inhibitory and excitatory inputs occur in a precise and stereotyped temporal sequence, which is called balanced inhibition, and serves as a mechanism for frequency selectivity, which contradicts the predictions of classical lateral inhibition models [36]. Further studies have shown that this inhibition is relatively stronger in frequency domains flanking the BF of the recording neuron, and in the sharpening of the frequency selectivity [35, 37–38]. However, the frequency selectivity had also been reported to be sharpened by lateral inhibition via an unconventional mechanism, which was attributed to a GABAergic synaptic input triggered by non-preferred stimuli which acting via indirect decreases in network activity, and is called network-level suppression [37]. In our present study, the FM component swept downward from the BF of the recording neuron, so it might activate the indirect network activity and worked as the non-preferred stimulus. Therefore, we speculate that the FM component would trigger the GABAergic synaptic transmission, suppressing the response to the CF component, and eventually leading to the sharpening of the selectivity to stimulation frequency, duration, and amplitude. A recent study has shown that the auditory-nerve responses could be suppressed by low-frequency bias tones, and this suppression originates from the reticular lamina motion and depends on the out hair cell stereocilia position [39]. The FM components could also serve as the low-frequency bias tones, indicating that the inhibition may start from the auditory nerve. Since the FM component was present after the CF component, the inhibitory effect of the FM component on the CF response may also serve as a backward masking mechanism, in which the tonal masker is subsequent to the test stimulus and suppress the responses to the test stimulus [40]. However, how the response to the later FM component can coincide with the earlier CF component and where this suppression originates from still needs further investigation.

We noticed that the SO neurons had slightly narrower BWs than DO neurons under CF stimulation conditions although not significant, indicating that the SO neurons were tend to have sharper frequency tuning tested with CF sounds only. And, in this case, the SO neurons may have even sharper frequency tuning when tested with CF-FM sounds since the FM component induced inhibitory input to the CF component. The BFs were kept unchanged when switching CF sounds to CF-FM sounds, possibly because that the frequencies of CF sounds were equal to neuron’s BF, which elicited the strongest excitatory input the neuron, so the effect of FM component would be relatively weaker.

### Biological significance of the present study

We first reported the SO and DO neurons in the IC of *Hipposideros armiger* [19]. Later on, these neurons were also recorded from the IC of *Hipposederos pratti* [22], indicating that the SO and DO response may be a common response property for hipposiderids. However, further studies on the other two genus, rhinolophids and *pteronotus parnellii*, are needed to confirm that the SO and DO response are of the basic response patters for the CF-FM bat. Recordings on the other auditory nucleus excepting the IC are also necessary for verifying the SO and DO response are general through all auditory pathway. Our present study showed that SO neurons had higher frequency selectivity than DO neurons under behavior-related CF-FM stimulation. In addition, the SO neurons that changed their selectivity when the stimulation was switched from CF to CF-FM sounds were tuned to the second harmonic of the echolocation signal. Previous studies also have shown that DO neurons had shorter recovery cycles and response latency than SO neurons [19, 20]. Taken together, the different auditory properties of the SO and DO neurons are responsible for the ability of bats to perform differential auditory functions during echolocation. It is possible that the SO neurons are required for velocity detection during the search and approach phases, because of their higher frequency selectivity and longer recovery cycle; while DO neurons may be suitable for target range and character determination during the terminal phase, because of their remarkable response to the FM component and shorter recovery cycle. Further studies using pair CF-FM sounds of different intervals are needed to verify the possible target range processing function of DO neurons.

## Supporting information

S1 Table The echolocation signal of the Pratt’s roundleaf bat, *Hipposideros pratti*. (DOCX)

## Abbreviations

BF: best frequency
BW: bandwidth
BS: both side symmetry
CF: constant frequency
DO: double-on
ED: excitatory duration
FM: frequency modulation
FTC: frequency tuning curve
HFN: high frequency side narrower
IC: inferior collicular
LFN: low frequency side narrower
MT: minimal threshold
PSH: post-spike hyperpolarization
PSHL: PSH latency
SO: single-on.
