# Supplementary material for: Behaviorally relevant frequency selectivity in single- and double-on neurons in the inferior colliculus of the Pratt’s roundleaf bat, Hipposideros pratti
Source: PLoS One. 2019 Jan 2;14(1):e0209446. doi: 10.1371/journal.pone.0209446 (PMC6314609; doi:10.1371/journal.pone.0209446)
Supplement: S1 Table — (DOCX) [file pone.0209446.s001.docx]

**Supplementary table 1. The echolocation signal of the Pratt’s roundleaf bat, *Hipposideros pratti*.**

| **Parameters** | **Min-Max** | **Median** | **Mean±SD** |
| --- | --- | --- | --- |
| **First harmonic** |  |  |  |
| CF (kHz)（N=75） | 26.3-38.2 | 30.9 | 31.2±1.6 |
| tFM (kHz)（N=75） | 18.8-27.8 | 23.3 | 22.7±2.2 |
| FM sweep range (kHz)（N=75） | 4.3-17.8 | 7.6 | 8.5±2.7 |
| Reduced percent in FM（N=75） | 13.7-46.6% | 25.3% | 27.1±7.6% |
| Duration (CF-FM/ ms)（N=36） | 2.5-7.3 | 4.7 | 4.7±1.4 |
| **Second harmonic** |  |  |  |
| CF_2_ (kHz)（N=75） | 59.3-60.9 | 60.8 | 60.3±0.7 |
| tFM_2_ (kHz)（N=75） | 42.7-48.8 | 45.6 | 45.4±1.4 |
| FM sweep range (kHz)（N=75） | 12-18.2 | 15 | 14.9±1.5 |
| Reduced percent in FM（N=75） | 19.7-29.9% | 24.7% | 24.6±2.3% |
| Duration (CF-FM/ ms)（N=75） | 6.5-11.1 | 9 | 8.9±1.1 |
| **Third harmonic** |  |  |  |
| CF_3_ (kHz)（N=74） | 84.8-91.0 | 89.4 | 89.2±1.3 |
| tFM_3_ (kHz)（N=74） | 74.1-86.5 | 83.6 | 82.6±3.3 |
| FM sweep range (kHz)（N=74） | 2.8-16.9 | 4.7 | 6.6±3.6 |
| Reduced percent in FM（N=74） | 3.1-18.6% | 5.4% | 7.4±4.0% |
| Duration (CF-FM/ ms)（N=49） | 4.2-7.7 | 5.4 | 5.6±1.2 |

N: Number of sound pulses. CF, constant frequency; FM, frequency modulation; tFM, terminal frequency of FM component.
